# Three Manual Noncommercial Methods to Prepare Equine Platelet-Rich Plasma

**DOI:** 10.3390/ani11061478

**Published:** 2021-05-21

**Authors:** Lorenzo G. T. M. Segabinazzi, Giorgia Podico, Michael F. Rosser, Som G. Nanjappa, Marco A. Alvarenga, Igor F. Canisso

**Affiliations:** 1 Department of Veterinary Clinical Medicine, College of Veterinary Medicine, University of Illinois Urbana Champaign, Urbana, IL 61802, USA; lgseg@hotmail.com (L.G.T.M.S.); gpodico@illinois.edu (G.P.); 2 School of Veterinary Medicine and Animal Science, São Paulo State University (UNESP), Botucatu 18618681, SP, Brazil; malvarenga@fmvz.unesp.br; 3 Department of Comparative Biosciences, College of Veterinary Medicine, University of Illinois Urbana Champaign, Urbana, IL 61802, USA; 4 Department of Pathobiology, College of Veterinary Medicine, University of Illinois Urbana-Champaign, Urbana, IL 61802, USA; mrosser2@illinois.edu (M.F.R.); Nanjappa@Illinois.edu (S.G.N.)

**Keywords:** platelet concentrate, horse, endometritis, blood byproduct, platelet viability

## Abstract

**Simple Summary:**

Platelet-rich plasma (PRP) is a popular therapy in human and veterinary medicine. This study compared three manual noncommercial methods to prepare PRP and its cooling. The three methods concentrated platelets and did not alter viability. Method 1 (i.e., involving double centrifugation) resulted in the greatest platelet concentrations, while method 3 (sedimentation) resulted in the lowest platelet concentration and greater contamination with white blood cells (WBC). Cooling increased platelet agglutination over time across methods and affected platelet viability in PRP obtained with method 3. It remains unknown whether the different methods and cooling would affect PRP’s clinical efficacy.

**Abstract:**

In light of PRP’s increasing popularity in veterinary practice, this study aimed to compare three manual methods to prepare and cool equine PRP. The blood of 18 clinically healthy mares was collected via venipuncture in a blood transfusion bag (method 1), blood tubes (method 2), and a syringe (method 3). In method 1, samples were double centrifuged; method 2 involved one centrifugation, and in method 3 the syringe was kept in an upright position to sediment for 4 h. After processing with three methods, PRP and platelet-poor plasma (PPP) were extracted and assessed for red (RBC) and white blood cell counts (WBC), platelet counts, and viability. In a subset of mares (*n* = 6), samples were processed with the three methods, and PRP was evaluated at 6 and 24 h postcooling at 5 °C. Method 1 resulted in the highest and method 3 in the lowest platelet concentration (*p* < 0.05), and the latter also had greater contamination with WBC than the others (*p* < 0.001). Platelet viability was similar across treatments (*p* > 0.05). Cooling for 24 h did not affect platelet counts in all methods (*p* > 0.05); however, platelet viability was reduced after cooling PRP produced by method 3 (*p* = 0.04), and agglutination increased over time in all methods (*p* < 0.001). The three methods increased (1.8–5.6-fold) platelet concentration in PRP compared to whole blood without compromising platelet viability. In conclusion, all three methods concentrated platelets and while cooling affected their viability. It remains unknown whether the different methods and cooling would affect PRP’s clinical efficacy.

## 1. Introduction

Platelet-rich plasma (PRP) is a biological byproduct derived from whole blood (WB) after removal of red blood cells (RBC) and complete or partial removal of white blood cells (WBC) to concentrate platelets in a small volume of plasma. It is traditionally used in human and veterinary medicine to treat orthopedic and dermatological diseases [1,2]. Recently, PRP also gained popularity to mitigate persistent breeding-induced endometritis (PBIE) [3,4] and improve fertility in mares with chronic degenerative endometritis [5] and barren mares [6,7,8]. The immunomodulatory properties of PRP were attributed to growth factors (e.g., hepatocyte growth factor) and cytokines (e.g., transforming growth factor β, CXCL8, and IL1β) present in the platelets [9,10]. In addition, the platelet granules contain antimicrobial peptides (e.g., RANTES and platelet factor 4) that may be beneficial against bacterial infections [11,12,13].

Automatized (e.g., AngelTM Cytomedix, Inc. Gaithersburg, MD, USA; and Restigen PRP^®^, Owl Manor, Warsaw, IN, USA) and manual protocols were described to prepare PRP for usage in clinical practice [14,15,16,17,18]. The first approach offers the advantage of being standardized and less human dependent; however, the high cost of acquiring the machines and supplies can discourage small practices or solo practitioners to routinely use PRP in clinical practice. Conversely, manual methods require no specialized equipment and can be affordable alternatives to prepare PRP.

The majority of manual protocols used in equine practice involve one or two centrifugations to concentrate platelets in a small volume of plasma (e.g., 2–5 mL) for injection in tendons or intra-articular treatment [14,19]. However, presumably for intra-uterine therapy, a higher volume (e.g., 10–60 mL) is needed to reach the endometrium’s entire surface [20]. In addition, PRP is often used immediately after its preparation at room temperature [21]. However, multiple infusions of PRP were proposed to maximize its efficacy [4], and the ability to cool down PRP for subsequent infusion the next day would facilitate its use in clinical practice. In human medicine, PRP is often stored up to seven days at room temperature (22 °C) [22]; however, this temperature can increase the risk of bacterial growth [23].

Therefore, this study’s objectives were to determine and compare three manual non-commercial methods to prepare and cool equine PRP. Specific endpoints assessed included cell composition, platelet counts, viability, and agglutination. The hypotheses of the present study were that the methods tested herein produce PRP with platelet concentration (2–9-fold in comparison to WB) with high viability, and cooling PRP has no detrimental effects on platelet viability.

## 2. Materials and Methods

### 2.1. Animals

The study was carried out from November 2019 to November 2020 at the College of Veterinary Medicine, University of Illinois Urbana-Champaign. A total of 18 light breed mares aged 5–16 years old (9.4 ± 3.0 years-old) and weighing 400–600 kg, were enrolled in the study. The mares belonged to the teaching/research herd of the University of Illinois Urbana-Champaign. All mares were deemed clinically healthy based on full physical examination and complete blood cell count immediately before enrollment in the study (as illustrated in Table 1). The mares were housed in individual stalls, fed with 5 kg of mixed alfalfa-grass hay 3 times a day, with free access to water and trace minerals. All mares were vaccinated and dewormed regularly, following the American Association of Equine Practitioners guidelines [24,25]. Mares had their feet trimmed and teeth floated following the Ag-guide for Agricultural animals following USDA recommendations. All animals were kept at the University of Illinois campus.

### 2.2. Experimental Design

**Figure 1:**
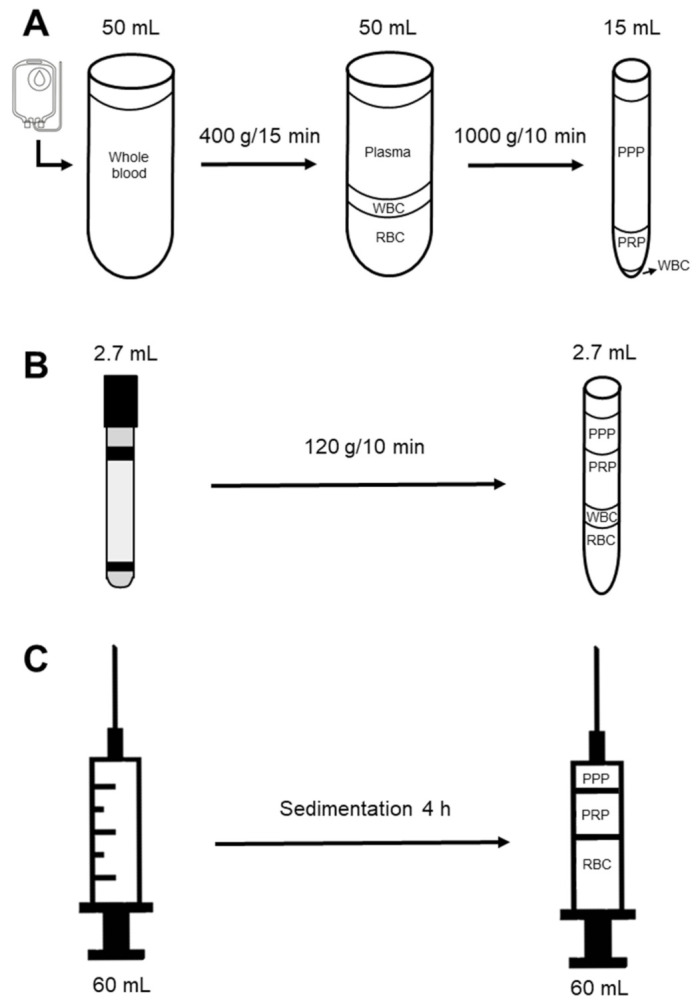
Diagram of three methods to prepare platelet-rich plasma (PRP) for intrauterine infusions in mares. (**A**) Method 1, blood was collected in a 150 mL blood transfusion bag and processed by 2-step centrifugation; (**B**) Method 2, blood was collected in 2.7 mL vacutainer tubes and processed by one-step centrifugation; (**C**) Method 3, blood was collected in a 60 mL syringe and PRP produced by sedimentation. Abbreviations: PPP, platelet-poor plasma; WBC, white blood cells; RBC, red blood cells.

All mares had blood samples sequentially collected in a blood collection bag (method 1), blood collection tubes (method 2), and a 60 mL syringe (method 3). After collection, an aliquot of WB from each method was submitted to the University of Illinois Clinical Pathology Laboratory for a complete blood cell count with an automated machine (Sysmex XT-2000 iV, Kobe, Japan). In addition, platelets from WB were stained and had viability assessed as described below. The WB obtained with each method was processed separately to harvest PRP and PPP as diagrammatically depicted below (as illustrated in Figure 1). In addition, a subset of mares (*n* = 6) was randomly selected and had PRP harvested and stored for 24 h in a refrigerator at 5 °C. At 6 and 24 h post cooled-storage, PRP was assessed for platelet counts, viability, and agglutination as described below. No cryoprotectants were added during cooling.

#### 2.2.1. Method 1

Blood was collected from each animal through a puncture of the jugular vein using an 18 G needle into a 150 mL blood transfusion bag (Fresenius Kabi AG, Bad Homburg, Germany) containing 21 mL citrate-phosphate-dextrose solution with adenine as an anticoagulant (CPD-A, Santa Cruz Biotechnology, Inc., Dallas, TX, USA) according to modified techniques previously described [4,14]. A total of 100 ml of WB was split into two 50 mL tubes and was centrifuged at 400× *g* for 15 min. The plasma was transferred into 15 mL conical tubes and centrifuged at 1000× *g* for 10 min. Both centrifugations were performed at room temperature, with deceleration force off. After the second centrifugation, 2.5 mL of plasma at the bottom of each tube was preserved and deemed as PRP, while the supernatant above the 2.5 mL was considered as PPP. The concentrations of platelets, WBC, and RBC were determined in WB, plasma after the first centrifugation, and PPP and PRP samples at University of Illinois Clinical Pathology Laboratory.

#### 2.2.2. Method 2

Both PRP and PPP were obtained after single centrifugation as described elsewhere [3]. Briefly, 32.4 mL (12 tubes) of WB was collected from each animal through a puncture of the jugular vein into 2.7 mL blood collection tubes containing 3.2% sodium citrate (BD Vacutainer^®^, Becton, Dickinson and Company Franklin Lakes, NJ, USA). Blood tubes were immediately centrifuged at 120× *g* for 10 min at room temperature. After centrifugation, the top third layer of plasma was deemed as PPP, while the remaining portion was considered as PRP.

#### 2.2.3. Method 3

Blood was collected through a puncture of the jugular vein using an 18 G needle into a 60 mL syringe prefilled with 7 mL of CPD-A as an anticoagulant. Right after collection, each syringe was wrapped in aluminum foil and placed in an upright position for 4 h. Subsequently, a 21 G butterfly catheter was connected to the syringe, and PPP and PRP were recovered by applying steady pressure to the syringe’s plunger in an upright position (as illustrated in supplementary file: video S1). The top 10 mL of plasma was considered PPP, and the remaining plasma until the sedimented blood cells was deemed PRP. Buffy coat was not visible in this method.

### 2.3. Cell Counts

Whole blood samples collected in 0.1% sodium citrate anticoagulant were submitted to the University of Illinois Clinical Pathology Laboratory for automated quantification of platelet, WBC, and RBC counts. The Sysmex XT-2000 iV analyzer (Sysmex) reported platelet counts obtained both by DC sheath flow (PLT-I) and fluorescence flow cytometry (PLT-O). Manual blood smear evaluation with platelet estimation was performed for each sample by a board-certified clinical pathologist. If the PLT-O and PLT-I measurements were discordant, then the value closer to the manual platelet estimate was used for statistical analysis.

During the manual counting, the presence and quantity of platelet clumping were graded as score 0, absence of platelet clumps; score 1, small platelet clumps identified in the hemocytometer chamber, but not within the squares used to obtain the platelet count; score 2, small platelet clumps (~2–5 platelets/clumps) occurring in low numbers throughout the chamber and within the squares used for counting; score 3, presence of low numbers of larger platelet clumps (~5–20 platelets/clump), or high numbers of small platelet clumps; score 4, presence of many large platelet clumps, which would invalidate a manual platelet estimate. In addition, manual RBC, WBC, and platelet counts were performed on all PRP and PPP samples. The same board-certified clinical pathologist performed all the assessments.

For RBC and WBC counts, a 1:100 dilution of PRP or PPP was made using 0.9% NaCl solution. Samples were mixed thoroughly and loaded into a hemocytometer. Once the chambers were loaded, the sample was allowed to sediment for 10 min in a humidified chamber. The average number obtained from counting both chambers was used for the statistical analysis. If the counts obtained from each side of the hemocytometer differed by >10%, the counts were rejected, and the procedure was repeated. For platelet counting, a 1:100 dilution of PRP or PPP was made using 1% ammonium oxalate solution; samples were mixed thoroughly and allowed to rest for at least 5 min before loading the hemocytometer. Hemocytometer preparation and cell counting were performed as described above, and platelet clumping was noted, if present. The criteria for platelet clumping assessed on the hemocytometer were those used for the blood smear.

### 2.4. Platelet Viability

Platelet viability was assessed using a full-spectrum detector-based (filterless) Cytek Aurora Flow Cytometer (Cytek Biosciences Inc, Fremont, CA, USA) adapted from protocols published elsewhere [26,27]. Immunolabeling of CD41/61, a platelet-specific antigen, was carried out with a primary (#MA5-28,370 Invitrogen, Life Technologies Corporation, OR, USA) and a secondary antibody conjugated with a fluorochrome (#P-852, Invitrogen, Life Technologies Corporation, Carlsbad, CA, USA). In addition, the association with zombie green (#423,112 Biolegend, San Diego, CA, USA), a dye that binds to cytoplasmic amines, was used to assess the plasma membrane integrity of platelets (onwards referred to as platelet viability). The working solution of zombie green was prepared with the dilution in PBS at a 1:100 ratio. Briefly, an aliquot of PRP or PPP was diluted 1:20 in Tyrode’s media (134 mM NaCl, 12 mM NaHCO3, 2.9 mM KCl, 0.34 mM Na2HPO4, 1 mM MgCl2, 10 mM HEPES; pH 7.4) deprived from calcium chloride. Thereafter, the sample was incubated with zombie green working solution (1:1) and anti-CD41/61 monoclonal mouse antibody (1:200) for 30 min at room temperature in the dark.

Samples were washed (1500× *g*, 15 min), resuspended in Tyrode’s media, and further incubated with polyclonal goat antimouse IgG (1:100) for 30 min at room temperature in the dark. Samples were washed (1500× *g*, 15 min), resuspended in 200 µL of the same Tyrode’s media, and immediately analyzed. This panel identified three different populations: (1) viable platelets (CD41/61+ with low zombie green intensity), (2) nonviable (damaged) platelets (CD41/61+ with high zombie green intensity), and (3) debris (CD41/61 negative with high and low zombie green intensity). The sample acquisition by Cytek Aurora flowcytometric analyzer was concluded when at least 1,000,000 fluorescent gated events or 150 µL of the sample were assessed. Single stain controls were used for compensation and unmixing the signals using SpectroFlow^®^ software. Heat-treated platelets (75 °C, 15 min) served as a positive control for nonviable platelets. Data were analyzed with the software FlowJo (FlowJo, V10.6.2, BD Biosciences, Franklin Lakes, NJ, USA); the percentage of events in each population was calculated, and manual compensation was applied as needed.

### 2.5. Enrichment Factor and Platelet Recovery and Loss

The enrichment factor was calculated for platelets, WBC, and RBC before and after centrifugation for PPP and PRP across methods and after the first centrifugation in method 1. The value obtained in WB was considered the absolute value, and an increase or decrease in platelets or blood cells was used to attain this factor. For instance, if a sample had 100 units of an analyte in WB and the plasma sample had 160 units, the enrichment factor was deemed 1.6. Conversely, if the plasma sample had 40 units, the enrichment factor was deemed 0.4. Platelet recovery was calculated by using WB total platelet counts. The amount of platelet recovered in PRP and losses in PPP were calculated and used for comparisons across methods. Plasma recovery (i.e., the ratio of the PRP volume to the WB volume harvested from the mares) was calculated by using WB total volume as the absolute value, and the final volume of PRP was obtained after processing by each method.

### 2.6. Data Analyses

The volume of PRP and PPP were normalized to 20 mL for comparisons across methods; specifically, the concentrations (number/µL) of platelets, WBC, and RBC were assessed; therefore, these results were multiplied by 1000 to obtain the concentration per mL, and then by 20 to obtain final amounts. This approach was adopted due to the heterogenous volume of WB harvested for each method and consequent variable concentration. The sample size was determined based on a parallel study involving the use of PRP in mares. Previous studies comparing methods to prepare PRP typically used 6–8 horses [14,15,16,17,18], while here, 18 horses were used for most endpoints and 6 horses for the cooling endpoints. Data analyses were carried out with GraphPad Prism 8.0.1. (GraphPad Prism 8.0.1., GraphPad Software, San Diego, CA, USA). The Gaussian distribution curves (platelets, WBC, RBC, enrichment factor, plasma recovery, platelet clumps, and viability) were evaluated using the Shapiro–Wilk normality test. Platelet, WBC, and RBC concentrations, platelet enrichment factor, plasma recovery, and platelet viability were evaluated by ANOVA followed by Tukey’s as a post hoc test. Platelet clumps were evaluated by Friedman’s test, followed by Dunn’s multiple comparisons. Significance was set at *p* ≤ 0.05 for all tests. The degree of linear correlation between platelet counting in the WB and PRP for each method was tested using Pearson’s coefficient correlation. Strong coefficient of correlation was defined as *r* > 0.7, and moderate 0.5 ≤ *r* ≤ 0.7, and weak correlation when *r* < 0.5.

## 3. Results

The platelet, WBC, and RBC concentrations of the 18 mares were not different in the WB across methods (*p* > 0.10) (as illustrated in Table 2). All three methods concentrated platelets at least 1.8–5.6-fold in comparison to that of WB. In addition, WBC and RBC concentrations were reduced 2.9–4.15-fold and 46.6–321-fold, respectively, in comparison to that of WB (*p* < 0.0001) (as illustrated in Table 3). The platelet concentration was the greatest in PRP obtained with method 1, followed by methods 2 and 3 (as illustrated in Table 2, *p* < 0.001).

Platelet enrichment was the greatest (5.6) in PRP prepared with method 1, followed by methods 2 (2.5) and 3 (1.8) (as illustrated in Table 2) (*p* < 0.0001). The enrichment factor was 1.6 for platelet concentration when compared with that of WB after first centrifugation in method 1. In addition, plasma obtained from the first centrifugation in method 1 had a similar platelet concentration compared to that of PRP obtained with method 3 (*p* = 0.27); however, it was lower than PRP obtained by methods 1 (*p* < 0.0001) and 2 (*p* < 0.0001) (as illustrated in Table 2). Concentrations of platelets in PPP were lower than PRP across methods (*p* < 0.0001) (as illustrated in Table 2). In addition, concentrations of platelets in PPP were lower than in WB for methods 1 and 2 (*p* < 0.0007), but not different in method 3 (*p* = 0.99) (as illustrated in Table 2).

**Figure 2:**
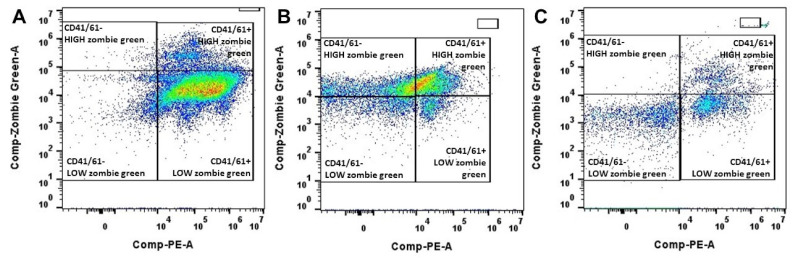
Representative density plot for flow cytometric analyses performed on PRP obtained by method 1 (**A**), method 2 (**B**), and method 3 (**C**). Platelets were identified with a primary (mouse monoclonal antibody anti CD41/61) and secondary antibody antimouse IgG conjugated with a fluorochrome (PE, R-phycoerythrin, X-axis) and their membrane integrity was assessed with zombie green (Y-axis). Right quadrants enclosed CD41/61 positive events, negative events, likely debris, with high or low zombie green signal. Method 1 consisted of collecting WB in a blood transfusion bag, centrifuging at 400× *g*/10 min, and then re-centrifuging at 1000× *g*/10 min. Method 2 consisted of one centrifugation at 120× *g*/10 min. Method 3 involved assessment of platelet concentration in PRP-3 and PPP-3.

**Figure 3:**
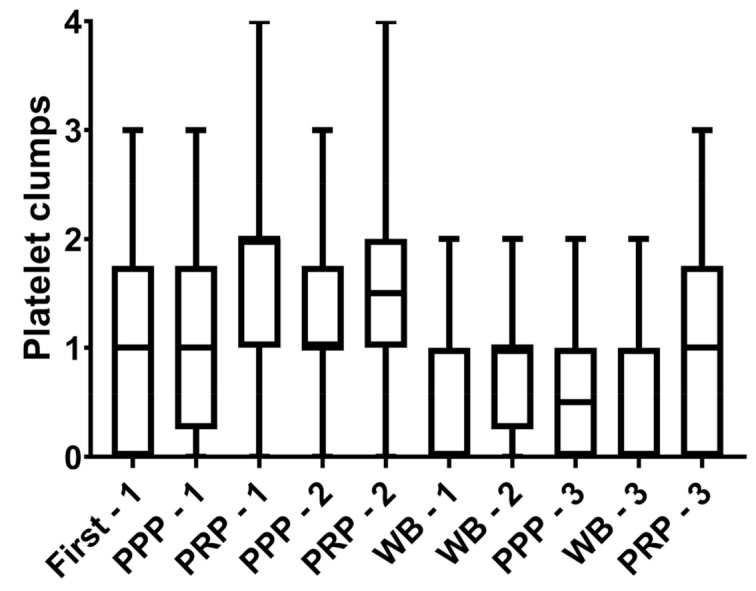
Assessment of platelets harvested from 18 mares by 3 methods to prepare PRP for intrauterine infusions in mares. Method 1 consisted of collecting whole blood (WB) in a blood transfusion bag, centrifugation of WB at 400× *g*/10 min (F-C), and centrifugation of plasma at 1000× *g*/10 min. Method 2 consisted of one centrifugation at 120× *g*/10 min. Method 3 consisted of collecting WB in a syringe and then letting it sediment in an upright position for 4 h. After processing, PRP and PPP were obtained across methods. Platelet clumps were assessed across methods. Clumps classification: score 0, absence of platelet clumps; **score 1**, small platelet clumps identified in hemocytometer chamber, but not within squares used to obtain platelet count; **score 2**, small platelet clumps (~2–5 platelets/clumps) occurring in low numbers throughout chamber and within squares used for counting; **score 3**, presence of low numbers of larger platelet clumps (~5–20 platelets/clump), or high numbers of small platelet clumps; **score 4**, presence of many large platelet clumps.

Platelet viability was similar in fresh WB and respective plasma samples (*p* = 0.97) (as illustrated in Table 2). Representative images of flow cytometric analyses of platelet viability in PRP are depicted in Figure 2. Strong and positive correlations were found between the number of platelets in the WB and the number of platelets recovered in PRP prepared by methods 2 (*r* = 0.75) and 3 (*r* = 0.73). However, a moderate correlation (*r* = 0.53) was observed between platelet concentration in WB and PRP produced by method 1. There were no changes in the occurrence of platelet clumps in WB and respective plasma samples across methods (*p* > 0.05, as illustrated in Figure 3).

The platelet recoveries in the PRP from methods 1, 2, and 3 were 56%, 81%, and 77%, respectively. In PPP, the platelet losses were 21%, 15%, and 13% using methods 1, 2, and 3, respectively. The recovery factor (i.e., the ratio of the PRP volume to the WB volume) of plasma recovered as PRP was different across methods (*p* < 0.0001; method 1, 10.5%; method 2, 33.1%; method 3, 27.2%).

The WBC was reduced (*p* < 0.0001) in all PPP and PRP samples, as well as in the plasma after the first centrifugation performed in method 1, when compared with that of WB (as illustrated in Table 3). However, PRP obtained by method 3 had greater WBC than plasma samples from methods 1 and 2 (*p* < 0.001, Table 3). The WBC was 1.9- and 71.0-fold reduced in the PRP produced by method 1 compared with the PRP from methods 2 and 3, respectively. Also, the PRP from method 2 had a 38.1-fold reduction in WBC counts than PRP produced by method 3. The concentration of RBC was also reduced (*p* < 0.0001) in all plasma samples compared with the respective WB samples (as illustrated in Table 3).

**Figure 4:**
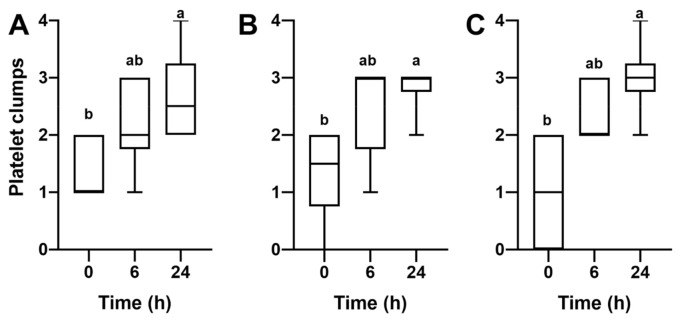
Presence of platelet clumps in PRP of six mares, having WB harvested and processed by 3 methods to prepare PRP for intrauterine infusions in mares and cooled-stored at 5 °C for up to 24 h. (**A**) Method 1 consisted of collecting WB in a blood transfusion bag, centrifugation of WB at 400× *g*/10 min (F-C), and centrifugation of plasma at 1000× *g*/10 min. (**B**) Method 2 consisted of one centrifugation at 120× *g*/10 min. (**C**) Method 3 consisted of collecting WB in a syringe and then letting it sediment in an upright position for 4 h. After processing (0 h), PRP was obtained across methods and cooled-stored at 5 °C for 6 and 24 h. Platelet, WBC, and RBC counts and platelet viability were assessed in fresh (0 h) and cooled-stored PRP at 6 and 24 h. Clumps classification: score 0, absence of platelet clumps; **score 1**, small platelet clumps identified in the hemocytometer chamber, but not within the squares used to obtain the platelet count; **score 2**, small platelet clumps (~2–5 platelets/clumps) occurring in low numbers throughout the chamber and within the squares used for counting; **score 3**, presence of low numbers of larger platelet clumps (~5–20 platelets/clump), or high numbers of small platelet clumps; **score 4**, presence of many large platelet clumps. Differences within column superscripts (^a,b^) indicate differences between groups (*p* < 0.05).

Platelet, WBC, and RBC counts, and platelet viability did not change after cooling storage (*p* > 0.05; Table 4) of PRP of six mares produced by methods 1 and 2. However, platelet viability was reduced at 24 h of cooled storage in PRP obtained by method 3 (as illustrated in Table 4). In addition, there was an increase in the occurrence of platelet clumps over time in PRP cooled-stored (*p* < 0.05, as illustrated in Figure 4).

## 4. Discussion

This study was set forth as the first to compare three manual noncommercial protocols used to prepare PRP and its cooling. The three methods were selected for having potential application in equine clinical practice. Previously, blood transfusion bags (450 mL) were used to prepare PRP for wound dressing and intrauterine infusion [4,14,16]; here, a smaller bag (150 mL), commonly used for blood collection and transfusion in dogs and cats, was used. Method 2 was used in clinical practice and in a couple of studies in equine practice [3,28]. Method 3 was used in practice but not studied in a controlled study.

Previously reported methods to prepare equine PRP involve centrifugation or the use of specialized automated machines to concentrate platelets [3,5,6,7,8,27,28,29,30,31,32,33,34]. Overall, PRP was applied as an adjuvant treatment for many equine musculoskeletal injuries (e.g., osteoarthritis, tendinitis, and laminitis) and skin wounds [14,29,30,31,32,33,34,35,36]. Double centrifugation methods involve placement of WB in blood tubes to concentrate platelets at 320–560 × 10^3^ of platelets/µL [29,31,32,33,34,35]. Other authors described a commercial method (E-PET^TM^) to obtain PRP for intra-articular injection in horses with osteoarthritis. Those authors reported platelet concentrations ranging from 540–950 × 10^3^ of platelets/µL. For musculoskeletal PRP treatments, the volume varies according to the site of injection from 2.5–5 mL [14,29,30,31,32,33,35,36]. Early studies involving PRP intrauterine infusions in mares applied a specialized blood fractionated machine (AngelTM Cytomedix) [6,7]. The volume of plasma recovered by this technology is much reduced compared with manual methods to the point that the authors of these reports diluted the PRP in PPP to bring the final volume up to 10 mL. The amount of WB needed to prepare PRP varies with the method used. In the two early reports, the authors did not include the amount of blood harvested, platelet concentration, or final counts obtained with the machine [6,7]. In a more recent study, the authors also used a commercially available device (Restigen PRP^®^) to obtain PRP. For this, 55 mL of WB was harvested in a 60 mL syringe containing 5 mL of anticoagulant from each mare to produce 6 mL of PRP, which was diluted in 9 mL of PPP to produce a final volume of 15 mL for intrauterine infusion [8]; these authors did not provide the platelet concentrations. In another study, using a manual method, 100 milliliters of WB were harvested in vacutainer tubes and centrifuged at 120× *g* for 10 min; after the first centrifugation, the lower 50% of the plasma was further centrifuged at 240× *g* for 10 min. This protocol yielded 20 mL of PRP (containing ≥ 250 × 10^3^ of platelets/µL, ~5 × 10^9^ platelets) [5]. In another study, 45 mL of WB was collected in 4.5 mL sodium citrate tubes and centrifuged once at 120× *g* for 10 min; the protocol resulted in 20 mL of PRP containing 7 ± 0.3 × 10^9^ platelets (354 ± 17 × 10^3^ of platelets/µL) [3]. In the present study, the total platelets obtained ranged from 4.8 ± 0.3 to 14.2 ± 0.9 × 10^9^ when normalized to 20 mL. Therefore, the three protocols investigated herein resulted in platelet concentrations consistent with other studies performing intrauterine infusions with PRP in mares [3,5].

All methods tested herein were sufficient to increase the plasma’s platelet concentration (1.8–5.6-fold) and reduce RBC and WBC as previously attained by other studies [3,17,18]. Studies suggest that increments of 3–9 folds in human platelet concentration compared to the WB is ideal [37]; however, the optimal platelet concentration is yet to be defined for clinical use in horses [3,14,38].

Method 1 resulted in PRP with platelet concentrations of 2.2- and 3.0-fold higher than methods 2 and 3. This finding could be because a larger volume of WB was used (3- to 4-fold greater) when compared with that of other methods, but this is more likely because 2-step centrifugation was used in method 1. The second centrifugation in method 1 applied a much greater g-force than the other methods, yet the platelet viability was not affected in PRP or PPP. Platelet concentration in PRP obtained by methods 2 and 3 was strongly correlated with the platelet concentration in WB, while this was not the case for method 1. These findings suggest that 2-step centrifugation may help standardize platelet recovery in comparison with that of method 2, which only uses 1-step centrifugation, and method 3 did not employ any imposed force other than the gravitational force.

The volume of WB harvested to produce PRP in this study is equivalent to other studies using PRP in horse joints and tendons [14,38,39] and for intrauterine infusion in mares [6,7]. Tendons have no lumen, and joints have reduced space, thus requiring highly concentrated platelets in a reduced volume of PRP. Conversely, the mare uterus has a large uterine lumen, therefore, presumably requiring a larger volume of PRP. In addition, mares susceptible to PBIE, the population to benefit the most from PRP therapy, are often older, multiparous, and have a large and pendulous uterus [40,41]. Generally, it is believed that intrauterine infusion should have a volume of 10–60 mL to allow the substance being infused to interact with the entire uterine lumen. In the present study, all three methods would meet this presumed ideal volume range.

Of interest, anticoagulants reported for harvesting WB to produce PRP in horses include sodium citrate, CPD-A, and acid-citrate-dextrose solution [3,5,6,7,8,28,34]. Herein CPD-A was used in methods 1 and 3, and sodium citrate in method 2. There is no data to suggest that either one of the anticoagulants used in the present study is superior to another; however, both were superior to acid-citrate-dextrose solution in two studies [42,43]. Certainly, in this study, the platelet viability and clumps were not affected by the method used.

While there appears to be no clear advantage of either method over another, some clinicians may feel more comfortable with one method versus another. Interestingly, plasma after the first centrifugation at 400× *g* in method 1 yielded 1.6-fold greater platelet concentration than the WB. This finding suggests that PRP may be prepared using 400× *g* centrifugation for 10 min in 50 mL conical tubes for use in clinical practice. This method would minimize the losses associated with the second centrifugation but would increase the volume. Large volume of PRP could represent an issue for tendons, joints, and uterus of maiden mares; thus, in these conditions, the second centrifugation is necessary.

In all three methods, RBC and WBC were reduced in all plasma samples when compared with that of WB. Contamination of WBC and RBC in PRP can contribute to platelet aggregation and activation [44]. In addition, removing RBC seems beneficial as their presence in excess can be detrimental to sperm if PRP was used for uterine infusion, thus limiting PRP’s use pre-breeding [45]. However, controversy exists over whether the presence of fresh WBC from WB could be beneficial for various equine diseases [46,47] The PRP produced by method 3 had greater WBC concentrations, likely because of the lack of centrifugation to forcefully push blood cells to the bottom, and the clinical relevance of this finding remains unclear. There is a scant body of evidence regarding the contribution of WBC in PRP to the healing process and inflammatory properties [48,49]. A beneficial effect was suggested to be due to their anti-infectious activity [50], and one report suggested that intrauterine infusion of WBC could eliminate bacterial contamination faster from the uterus of mares susceptible to PBIE [46]. However, it is unknown whether including fresh WBC from WB in PRP is beneficial for the treatment of PBIE in mares. In addition, contamination with WBC is not desired in PRP for musculoskeletal injuries [47,51,52].

The processing time for the methods tested in the present study was ~120 min for method 1, ~30 min for method 2, and ~240 min for method 3. Although method 3 required more time to be done, this method can be performed in ambulatory conditions, where the clinician may not have a centrifuge available. Four hours of sedimentation was chosen based on a pilot study. The results of the pilot study suggested that 4 h was the minimal time necessary for RBC sedimentation and to minimize RBC and WBC contamination, and also because if the PRP is being used for intrauterine infusion, four hours is the minimum time that a mare could be flushed after breeding without having compromised pregnancy rates [53] and to have the uterus infused with PRP. Therefore, method 3 can be an alternative protocol to prepare PRP in field conditions.

Platelets have a short lifespan, and in human medicine, they often are stored at room temperature, which enhances the chance of bacterial contamination [23]. Therefore, cold-storage of PRP has the potential to be an alternative to maintain platelet viability and reduce bacterial growth in PRP [54]. Although in one equine study, there were no platelet contamination or losses in platelet counts in PRP stored for up to seven days at room temperature (22 °C) on a rocker, the technique used for the preservation used a piece of specialized research equipment not available in clinical practice [17]. Also, the authors reported a reduction in platelet function over time [17]. In the present study, platelet counting and viability did not change up to 24 h of cooled-storage at 5 °C in PRP produced by methods 1 and 2. However, there was a reduced number of viable platelets in PRP cooled-stored for 24 h in PRP obtained by method 3. It is unclear why PRP produced by method 3 had lower platelet viability after cooled-storage, but this could be due to the greater contamination with WBC and RBC in PRP produced with method 3 when compared with that of methods 1 and 2. The presence of increased WBC in PRP can affect the metabolic activity of platelets and increase lactate production and glucose consumption, which can undergo wastage in the quality of PRP [44]. Other authors reported no changes in platelet counting in equine [55] or human [56] platelet concentrate after cooled-storage, but they did not report the concentration of RBC or WBC in their PRP.

Although the number of mares used for harvesting blood and processing PRP was greater than previous studies [14,15,16,17,18], there was a great range in age and breeds. In contrast, it is unknown if breed or age affects platelet concentrations, and the wide range of mares used herein reflected the efficacy of the methods tested in the present study to produce PRP in a wide range of horse populations. Also, while only six mares were used to cool down PRP, we believe that this subset of mares was representative of the whole group of mares. However, it is possible that different results may be obtained if a larger group of horses was used in the cooling segment of the study. Another limitation of the present study is that the clinical efficacy of the methods was not tested in mares.

## 5. Conclusions

In conclusion, the three manual methods were assessed to prepare PRP concentrated platelets. Method 1 (i.e., involving double centrifugation) resulted in the greatest platelet concentrations, while method 3 (sedimentation) resulted in the lowest platelet concentration and greater contamination with WBC. Cooling PRP did not compromise platelet viability for methods 1 and 2. However, cooling PRP prepared with method 3 decreased platelet viability. Also, cooling PRP without cryoprotector increased platelet agglutination over time and across methods. In vivo studies are warranted to assess the clinical efficacy of PRP obtained with the three methods and to determine the role of WBC and RBC contamination and platelet agglutination during cooling.

## Figures and Tables

**Table 1:** Mare signalment and platelet and blood cell counting immediately pre-enrolment.

Mare	Age	Breed	Weight (kg)	Blood Counts		
				Platelets (×10^3^/µL)	White Blood Count (×10^3^/µL)	Red Blood Count (×10^6^/µL)
1	5	Quarter Horse	480	120	7.0	7.2
2	6	Quarter Horse	470	116	6.6	6.5
3	9	Thoroughbred	540	125	5.0	6.4
4	8	Arabian	400	150	5.2	7.6
5	12	Paint Horse	520	152	6.0	7.8
6	6	Paint Horse	500	168	5.2	8.1
7	9	Standardbred	500	130	5.5	8.1
8	8	Standardbred	580	124	6.9	9.3
9	12	Standardbred	600	120	6.0	6.8
10	8	Quarter Horse	450	135	5.5	7.2
11	13	Thoroughbred	500	102	5.0	6.2
12	7	Arabian	400	118	6.5	6.7
* Subset (*n* = 6)						
13	16	Quarter Horse	480	172	5.1	6.1
14	14	Quarter Horse	520	112	5.6	7.5
15	10	Standardbred	580	106	5.4	6.8
16	11	Arabian	430	100	5.3	6.5
17	7	Saddlebred	510	138	5.9	6.1
18	8	Arabian	400	124	6.1	6.3

* The subset of mares used to harvest PRP for cooling.

**Table 2:** Assessment of platelets harvested from 18 mares by 3 methods to prepare PRP for intrauterine infusions in mares. Method 1 consisted of collecting whole blood (WB) in a blood transfusion bag, centrifugation of WB at 400× *g*/10 min (F-C), and centrifugation of plasma at 1000× *g*/10 min. Method 2 consisted of one centrifugation at 120× *g*/10 min. Method 3 consisted of collecting WB in a syringe and then letting it sediment in an upright position for 4 h. After processing, PRP and PPP were obtained across methods. Platelet concentration, viability, enrichment factor, and the number of clumps were assessed across methods.

		Concentration (×10^3^/µL)		Viability (%)		Enrichment Factor (%)
		Mean ± SD	Range	Mean ± SD	Range	
**Method 1**	**WB**	125.8 ± 27.6 ^d^	92–188	94.5 ± 10	60–99	-
	**F-C**	200.6 ± 60.2 ^c^	110–345	-	-	1.59
	**PPP**	44.2 ± 31.1 ^e^	15–130	85.9 ± 15	57–98	0.35
	**PRP**	709.4 ± 159.8 ^a^	425–960	92.5 ± 8	72–99	5.64
**Method 2**	**WB**	130.0 ± 18.4 ^d^	102–168	87.9 ± 16	50–99	-
	**PPP**	62.3 ± 23.7 ^e^	40–125	69.6 ± 24	53–99	0.37
	**PRP**	327.5 ± 64.2 ^b^	230–460	87.5 ± 15	55–98	2.52
**Method 3**	**WB**	129.6 ± 238 ^d^	80–172	85.6 ± 15	52–99	-
	**PPP**	105.9 ± 44.0 ^d^	45–180	84.1 ± 17	58–99	0.82
	**PRP**	239.4 ± 64.5 ^c^	180–435	86.8 ± 15	59–99	1.85

Clumps classification: **score 0**, absence of platelet clumps; **score 1**, small platelet clumps identified in the hemocytometer chamber, but not within the squares used to obtain the platelet count; **score 2**, small platelet clumps (~2–5 platelets/clumps) occurring in low numbers throughout the chamber and within the squares used for counting; **score 3**, presence of low numbers of larger platelet clumps (~5–20 platelets/clump), or high numbers of small platelet clumps; **score 4**, presence of many large platelet clumps. Different within columns superscripts (^a,b,c,d,e^) indicate differences between groups (*p* < 0.05).

**Table 3:** WBC and RBC counts in WB, PRP, and PPP of 18 mares, having WB harvested and processed by three methods to prepare PRP for intrauterine infusions in mares. Method 1 consisted of collecting WB in a blood transfusion bag, centrifugation of WB at 400× *g*/10 min (F-C), and centrifugation of plasma at 1000× *g*/10 min. Method 2 consisted of one centrifugation at 120× *g*/10 min. Method 3 consisted of collecting WB in a syringe and then letting it sediment in an upright position for 4 h. After processing, PRP and PPP were obtained across methods, and WBC and RBC counted.

		White Blood Cell			Red Blood Cell		
		Concentration (×10^3^/µL)		Enrichment Factor (%)	Concentration (×10^6^/µL)		Enrichment Factor (%)
		Mean ± SD	Range		Mean ± SD	Range	
**Method 1**	**WB**	5.9 ± 1.0 ^a^	4.4–7.1	-	6.4 ± 8.7 ^a^	5.1–7.8	-
	**F-C**	0.043 ± 0.018 ^bc^	0.02–0.08	0.007	0.058 ± 0.05 ^b^	0.02–0.1	0.009
	**PPP**	0.014 ± 0.009 ^c^	0.01–0.04	0.002	0.020 ± 0.02 ^b^	0.01–0.07	0.003
	**PRP**	0.027 ± 0.017 ^c^	0.01–0.06	0.005	0.042 ± 0.04 ^b^	0.03–0.1	0.007
**Method 2**	**WB**	5.7 ± 1.2 ^a^	4.5–7.0	-	7.3 ± 8.9 ^a^	6.2–9.3	-
	**PPP**	0.04 ± 0.03 ^c^	0.01–0.08	0.007	0.069 ± 0.07 ^b^	0.06–0.2	0.009
	**PRP**	0.05 ± 0.04 ^c^	0.01–0.2	0.009	0.083 ± 0.07 ^b^	0.02–0.2	0.011
**Method 3**	**WB**	5.5 ± 0.9 ^a^	4.3–7.2	-	6.4 ± 7.4 ^a^	4770–7500	-
	**PPP**	0.8 ± 1.1 ^b^	10–3180	0.15	0.13 ± 0.12 ^b^	22–400	0.020
	**PRP**	1.9 ± 1.7 ^b^	360–4740	0.35	0.11 ± 0.1 ^b^	4–305	0.017

Column with different superscripts (^a,b,c^) indicate differences between groups (*p* < 0.05).

**Table 4:** Platelet, WBC, and RBC counts, and platelet viability in PRP of 6 mares, having WB harvested and processed by 3 methods to prepare PRP for intrauterine infusions in mares and cooled-stored at 5 °C for up to 24 h. Method 1 consisted of collecting WB in a blood transfusion bag, centrifugation of WB at 400× *g*/10 min (F-C), and centrifugation of plasma at 1000× *g*/10 min. Method 2 consisted of one centrifugation at 120× *g*/10 min. Method 3 consisted of collecting WB in a syringe and then letting it sediment in an upright position for 4 h. After processing (0 h), PRP was obtained across methods and cooled-stored at 5 °C for 6 and 24 h. Platelet, WBC, and RBC counts, and platelet viability were assessed in fresh (0 h) and cooled-stored PRP at 6 and 24 h.

	Time (h)	Platelet (×10^3^/µL)		Platelet Viability (%)		WBC (×10^3^/µL)		RBC (×10^6^/µL)	
		Mean ± SD	Range	Mean ± SD	Range	Mean ± SD	Range	Mean ± SD	Range
**Method 1**	**0**	587.8 ± 133	455–825	81.7 ± 9	70–96	2.58 ± 3.2	0.5–5.0	0.04 ± 0.03	0.008–0.10
	**6**	510.3 ± 102	392–650	55.2 ± 12	39–74	1.25 ± 1.4	0.5–4.0	0.05 ± 0.03	0.02–0.09
	**24**	495.7 ± 74	412–582	52.3 ± 13	32–72	0.75 ± 0.3	0.5–1.0	0.04 ± 0.02	0.02–0.07
**Method 2**	**0**	508.7 ± 64	450–625	85.9 ± 9	82–98	2.83 ± 1.6	1.0–5.0	0.20 ± 0.30	0.03–0.73
	**6**	474.5 ± 102	400–615	61.1 ± 15	36–88	2.83 ± 1.3	2.0–5.0	0.10 ± 0.07	0.04–0.19
	**24**	448.2 ± 74	375–575	54.6 ± 19	27–83	1.25 ± 0.6	0.5–2.0	0.07 ± 0.05	0.03–0.15
**Method 3**	**0**	451.2 ± 116	365–610	89.3 ± 6 ^a^	74–98	47.17 ± 25.9	22.0–85.0	0.12 ± 0.11	0.03–0.31
	**6**	424.5 ± 94	330–590	60.1 ± 19 ^ab^	36–79	48.17 ± 19.0	22.0–67.0	0.12 ± 0.10	0.03–0.27
	**24**	368.7 ± 33	320–417	46.9 ± 21 ^b^	26–78	36.50 ± 26.4	11.0–88.0	0.14 ± 0.13	0.01–0.35

Differences within column superscripts (^a,b^) indicate differences between groups (*p* < 0.05).

## Data Availability

The original contributions presented in the study are included in the article/Supplementary Materials. Further inquiries can be directed to the corresponding author/s.

## Supplementary Materials

The following are available online at https://www.mdpi.com/article/10.3390/ani11061478/s1, Video S1: Harvesting PRP in Method 3.
